# Multilevel ordinal model for CD4 count trends in seroconversion among South Africa women

**DOI:** 10.1186/s12879-020-05159-4

**Published:** 2020-06-23

**Authors:** Zelalem G. Dessie, Temesgen Zewotir, Henry Mwambi, Delia North

**Affiliations:** 1 School of Mathematics, Statistics and Computer Science, University of KwaZulu-Natal, Durban, South Africa; 2 College of Science, Bahir Dar University, Bahir Dar, Ethiopia

**Keywords:** Cumulative logit, Factor analysis, Latent variables, Non-proportional odds models, Proportional odds models, Quality of life

## Abstract

**Background:**

Ordinal health longitudinal response variables have distributions that make them unsuitable for many popular statistical models that assume normality. We present a multilevel growth model that may be more suitable for medical ordinal longitudinal outcomes than are statistical models that assume normality and continuous measurements.

**Methods:**

The data is from an ongoing prospective cohort study conducted amongst adult women who are HIV-infected patients in Kwazulu-Natal, South Africa. Participants were enrolled into the acute infection, then into early infection subsequently into established infection and afterward on cART. Generalized linear multilevel models were applied.

**Results:**

Multilevel ordinal non-proportional and proportional-odds growth models were presented and compared. We observed that the effects of covariates can’t be assumed identical across the three cumulative logits. Our analyses also revealed that the rate of change of immune recovery of patients increased as the follow-up time increases. Patients with stable sexual partners, middle-aged, cART initiation, and higher educational levels were more likely to have better immunological stages with time. Similarly, patients having high electrolytes component scores, higher red blood cell indices scores, higher physical health scores, higher psychological well-being scores, a higher level of independence scores, and lower viral load more likely to have better immunological stages through the follow-up time.

**Conclusion:**

It can be concluded that the multilevel non-proportional-odds method provides a flexible modeling alternative when the proportional-odds assumption of equal effects of the predictor variables at every stage of the response variable is violated. Having higher clinical parameter scores, higher QoL scores, higher educational levels, and stable sexual partners were found to be the significant factors for trends of CD4 count recovery.

## Background

Human Immunodeficiency Virus (HIV) infection causes a weakening of the immune system ultimately leading to the development of Human Immunodeficiency Syndrome (AIDS) in the vast majority of infected persons if left untreated. One of the key biomarkers which is a predictor of progression to AIDS, as well as a means of monitoring a combination of antiretroviral therapy (cART) is the CD4 cell count. Low CD4 counts are associated with a greater risk of patients developing opportunistic infections, which may then progress to advanced disease stage [1, 2]. The use of cART generally results in the suppression of viral load replication and hence increased levels of CD4 cells. However, responses of the patients to therapy have heterogeneous behavior and complexity. Here, we use a multilevel longitudinal ordinal model to examine factors associated with adverse events (initial status and rate of change) of HIV infected patients.

Though the use of mixed models to accommodate hierarchical observations, has increased in medical research over the past several years, many researchers appear to have restricted their attention to multilevel *linear* models. These hierarchical mixed models assume that continuous variables are normally distributed within clusters and subjects. But very often medical research outcome measures are on an ordinal scale. The advantage of ordinal longitudinal model is that they account for floor and ceiling effects of the outcome variable [3, 4]. Accordingly, incorrect conclusions and biased estimates can be easily derived if the severity of HIV/AIDS is treated as continuous instead of ordinal [5]. Thus, we propose a multilevel growth model that may be more suitable for medical ordinal longitudinal outcomes.

Many researchers have carried out studies on the patterns of CD4 count changes or the obvious possible correlation among successive CD4 cell counts of HIV patients using quantile regression models [6–9] and generalized linear mixed-effects model [10–16]. In contrast, the present work aims at modelling of longitudinal adverse events (ordinal outcome) of HIV/AIDS in the presence of a hierarchical data structure. This is an important point that has not been considered in most HIV/AIDS cohort studies particularly in Sub-Saharan Africa. Furthermore, multilevel modeling for longitudinal ordinal responses, which is somewhat of new development in the analysis of medical data, tries to avoid the arbitrariness of assumptions involved when assigning the numerical scores through the use of cumulative probabilities in place of response probabilities for each category [17].

The goals of this paper are thus threefold. First, we seek to establish the long-time trend of adverse events of HIV infected patients using the generalized multilevel ordinal model. Second, we seek to fit and compare two frameworks of multilevel growth curve models with ordinal outcomes; namely the non-proportional-odds and the proportional-odds models, to avoid the fact that proportional odds assumption is used due to its simplicity and not because that is what best describes the data. Finally, we seek to justify that multilevel ordinal analysis is appropriate for our cohort data using intra-class correlation.

## Methods

### Study population

The data is from an ongoing prospective cohort study from CAPRISA. The original study, which started in August 2004, aimed at documenting acute infection with an extensive follow up to determine the natural history of the HIV-1 subtype C infection [18]. It was conducted at the Doris Duke Medical Institute (DDMRI) situated at the Nelson R. Mandela School of Medicine of the University of KwaZulu Natal in Durban, South Africa. Patients were recruited at two sites: an urban site in the city of Durban and a rural site in Vulindlela. In this study, patients who seroconverted during the HIV uninfected stage of CAPRISA 002 and other CAPRISA 004 and 008 trials [19, 20], were enrolled into the acute HIV Infection phase, and then followed-up during chronic infection and on cART. Further information about these ongoing prospective HIV cohort studies, including women’s eligibility criteria, were reported in [18, 21, 22]. Finally, two hundred and nineteen (219) participants were included in the study.

### Variables and measurements

Once HIV diagnosis was confirmed, participants were followed-up for a maximum of 13 years at the time of this analysis. CAPRISA initially enrolled HIV-negative (phase I) women into different study cohorts. Then seroconverted women were enrolled into a sequence of phases: acute infection (i.e. phase II: this was taken as follow up time within 0–3 months after infection), early infection (i.e. phase III: the time period during which the patients were followed up between 3 to-12 months’ post infection), established infection (i.e. phase IV: It started from 12 months’ post infection and ended once the patient has initiated antiretroviral therapy) and on cART (phase V: the patient was on ART- in this study, it was initiated when the CD4 cell count was below 500 cells/mm^3^). For the purpose of this study, samples for clinical attributes, virological, and immunological (such as CD4 cell count, viral load, RBC parameters, WBC parameters, blood chemistry, etc.) were measured at each visit (phase II-V).

In line with the World Health Organization (WHO) immunological classification criteria, we have categorized CD4 count to ordinal WHO classifications. These classification are defined as normal (CD4 > 500 cells/mm3), mild (350 < CD4 < 500), advanced (200 < CD4 < 350) and severe (CD4 < 200 cells/mm3) immunosuppression [23]. This WHO classification is a standard used by medical practitioners and health workers to monitor HIV infected patients.

The effect of several prognostic factors on the adverse events (initial status and slope value) of HIV infected patients, was investigated. The prognostic factors include (1) cART initiation, (2) demographics: age, marital status, educational level, and sex under the influence of alcohol; (3) OI: hypertension and tuberculosis; (4) clinical parameters: WBC components (neutrophils, lymphocyte count, monocytes, eosinophils count, and leucocyte count), Blood chemistry (sodium, chloride, calcium, ALT, AST, total protein and LDH), RBC parameters (Hb, RDW, MCH, MCV, MCHC, and hematocrit) (5) QoL domain scores. Detailed information about the above-mentioned covariates was given in [21, 22, 24].

### Statistical methods

Since the dataset has numerous clinical parameters, we used the factor analysis (FA) methods to minimize and group the parameters. Exploratory FA was done by creating the principal components of the original variables and then creating the eigenvectors. By using the Kaiser-criterions, an eigenvector with eigenvalues greater than 1 were kept [25]. A maximum likelihood extraction method with varimax rotation was used. Factor loadings describe the relationship of each clinical variable with each factor. The Factor loading is considered weak if less than 0.4, moderate if 0.4–0.6, and strong if greater than 0.6 [26]. Each observation was assigned a score for each rotated factor based on the loadings of the patient’s original variable levels.

Correlogram analysis was used to see the relations between the degree of severity and clinical attributes. Box plot was used to visualize the distribution of socio-demographic and clinical variables by the four grouping categories of CD4 counts.

### Generalized multilevel ordinal linear model

Explanatory models for ordinal response variable collected during a single time frame have been previously reviewed by Agresti [27], O’Connell [28], and Bender and Benner [29]. Such work was adapted to fit the needs of a hierarchical context. To mention a few, Fielding et al. [30] and O’Connell and Doucette [31] presented the application of the generalized multilevel ordinal model to educational data using the distribution of the latent variable. However, the generalized multilevel ordinal model is somewhat of underutilized method in clinical and epidemiological research studies.

When data are collected over time, methodologies for the handling of ordinal outcomes need to be combined with methods that address the multilevel nature of longitudinal data. Thus, event history data have a two-level hierarchical structure with repeated measures (level 1) nested within individuals (level 2). In the current work, a two-level ordinal analysis is applied. Suppose ordered values, k = 1, 2, …, K is assigned to a latent variable Y_ti_ related with level one unit ***t*** nested within level two unit *i*. The level two units consist of patient’s characteristics while level one units of longitudinally measured factors. The multilevel ordinal logistic models cumulative probabilities of the response variable (i.e., CD4 categories) rather than category probabilities using the logit link function as shows below:

1 $$ \mathrm{Log}\left(\frac{Prob\left({Y}_{ti}\le k\right)}{1- Prob\left({Y}_{ti}\le k\right)}\right)=\mathrm{logit}\left( Prob\left({Y}_{ti}\le k\right)\right)={\delta}^{(k)}+{X}_{ti}^{\prime}\beta +{u}_{ti} $$

Where k = 1, 2, …, K-1; ***δ***^(***k***)^, are the K-1 intercept terms to model the marginal frequencies in the K ordered categories; ${\boldsymbol{X}}_{\boldsymbol{ti}}^{\prime }$ a known matrix associated with the fixed effect **β** and ***u***_***ti***_ are random effects that are assumed to follow a multivariate-normal distribution with mean zero vector and variance **D**.

In the current study, the immunological state labeling is 1 for normal, 2 for mild, 3 for advanced, and 4 for severe immunosuppression. In this representation, a positive value of the coefficient β implies that higher values of the associated covariate are associated with a higher probability of being in smaller-numbered categories, i.e., at better immunological state.

A multilevel ordinal proportional-odds-model extension is a multilevel growth ordinal non-proportional-odds-model, which is a more widely and flexibly utilized model when the proportionality assumption of the effects of the predictor variables on the outcome variable does not hold. In this case, our ordinal outcomes variable will be modeled as.

2 $$ \mathrm{Log}\left(\frac{Prob\left({Y}_{ti}\le k\right)}{1- Prob\left({Y}_{ti}\le k\right)}\right)=\mathrm{logit}\left( Prob\left({Y}_{ti}\le k\right)\right)={\delta}^{(k)}+{\left({X}_{ti}^{\ast}\right)}^{\prime }{\beta}_c+{X}_{ti}^{\prime}\beta +{u}_{ti} $$

**x**^**∗**^_ti_ representing the vector containing the predictor variables for which proportional-odds is not assumed.

Estimation of both multilevel ordinal proportional-odds and non-proportional-odds model was carried out under a Bayesian framework through Markov chain Monte Carlo methods using MlwiN and SAS (NLMIXED code). The choice of Markov chain Monte Carlo method as the estimation technique over the commonly used marginal and penalized-quasi-likelihood procedure was to avoid the linearization of the logit, which tends to provide unreliable results [3, 32].

### Proportional odds assumption

The effect of each factor is assumed to be equal across the outcome categories. Relaxing this assumption Hedeker and Mermelstein [33] presented a multilevel ordinal non-proportional-odds model. A likelihood ratio test was carried out to assess the validity of the proportional odds assumption.

### Intra-class correlation (ICC)

For ordinal growth models, it is frequently of interest to state a proportion of total cluster level variations in terms of ICC. The intra-class correlation shows the proportion of total variance that is explained by group-level (i.e. level two: individuals) and is stated as $ICC={\sigma}_v^2/\left({\sigma}_v^2+{\sigma}^2\right)$, where ${\sigma}_v^2$ representing the variance of the patient-level effects (ie. The variance of level two) and *σ*^2^ representing the variance of level one (ie. the variance of the adverse events over time). For an ordinal multilevel model, the level one variance equals to the variance of the logistic model equal to *π*^2^/3 [34]. If the estimated ICC is high, there is evidence of a contextual effect on the outcome and which can be explored by including explanatory variables at each level.

## Results

### Baseline characteristics of the study population

The distribution of demographic and other clinical variables at baseline defined as the follow-up time within 0–3 months after the infection, is presented in Table 1. All patients were black women (*n* = 219), with a median age of 25 years (Interquartile range, IQR:22–30). The majority of patients were not with anemia (208; 95.0%), not co-infected with TB (201; 91.8%), married or with stable sex partners (174; 80%), and obese or overweight (137; 62.8%). Over half (153; 69.9%) reported having completed Grades 11 of schooling. Considering the immunological outcome, 40.6 and 36.5% of patients were having an baseline CD4 cell count of 350–499 and ≥ 500 cells/mm3, respectively. The viral load of patients ranged from 1.47 to 6.81 with a median of 4.46 (IQR:3.84–5.06) log10 copies/ml. The follow-up time of the patients ranged from 0-year to 13.13 years with a median of 2.12 (IQR:0.67–5.86) years (Table 1).

**Table 1:** Baseline Socio-demographic and clinical characteristics in the CAPRISA 002 trials

Variables	Count (percentage)
**Educational Status, n (%)**	
≤ Grade 8	16(7.31)
Grade 9–10	50 (22.8)
≥ Grade 11	153 (69.9)
**Marital Status, n (%)**	
Single/no sexual partner	34 (15.5)
Married/stable sexual partner	174 (79.5)
Many sexual partners	11 (5.0)
**Contraceptive use, n (%)**	
No	40 (18.3)
Yes	179 (81.7)
**Anemia, n (%)**	
No	208 (95.0)
Yes	11 (5.0)
**TB Co-infected, n (%)**	
No	201 (91.8)
Yes	18 (9.2)
**Immunological state, n (%)**	
< 200 CD4 cells/mm3	9 (4.1)
200–349 CD4 cells/mm3	41 (18.7)
350–499 CD4 cells/mm3	89 (40.6)
≥ 500 CD4 cells/mm3	80 (36.5)
**Age Categories, n (%)**	
18–20 years	29 (13.2)
21–39 years	178 (81.3)
≥ 40 years	12 (5.5)
**BMI Categories, n (%)**	
Underweight	5 (2.3)
Healthy weight	76 (34.9)
Overweight/Obese	137 (62.8)
**Sex act under the influence of alcohol, n (%)**	
No	197 (90.0)
Yes	22 (10.0)
**Follow-up time in years, Median (IQR)**	2.12 (0.67–5.86)
**Age, Median (IQR)**	25.0 (22.0–30.0)
**Log VL, Median (IQR)**	4.46 (3.84–5.06)

### Relationship between baseline hematological parameters and CD4 cell count

**Fig. 1:**
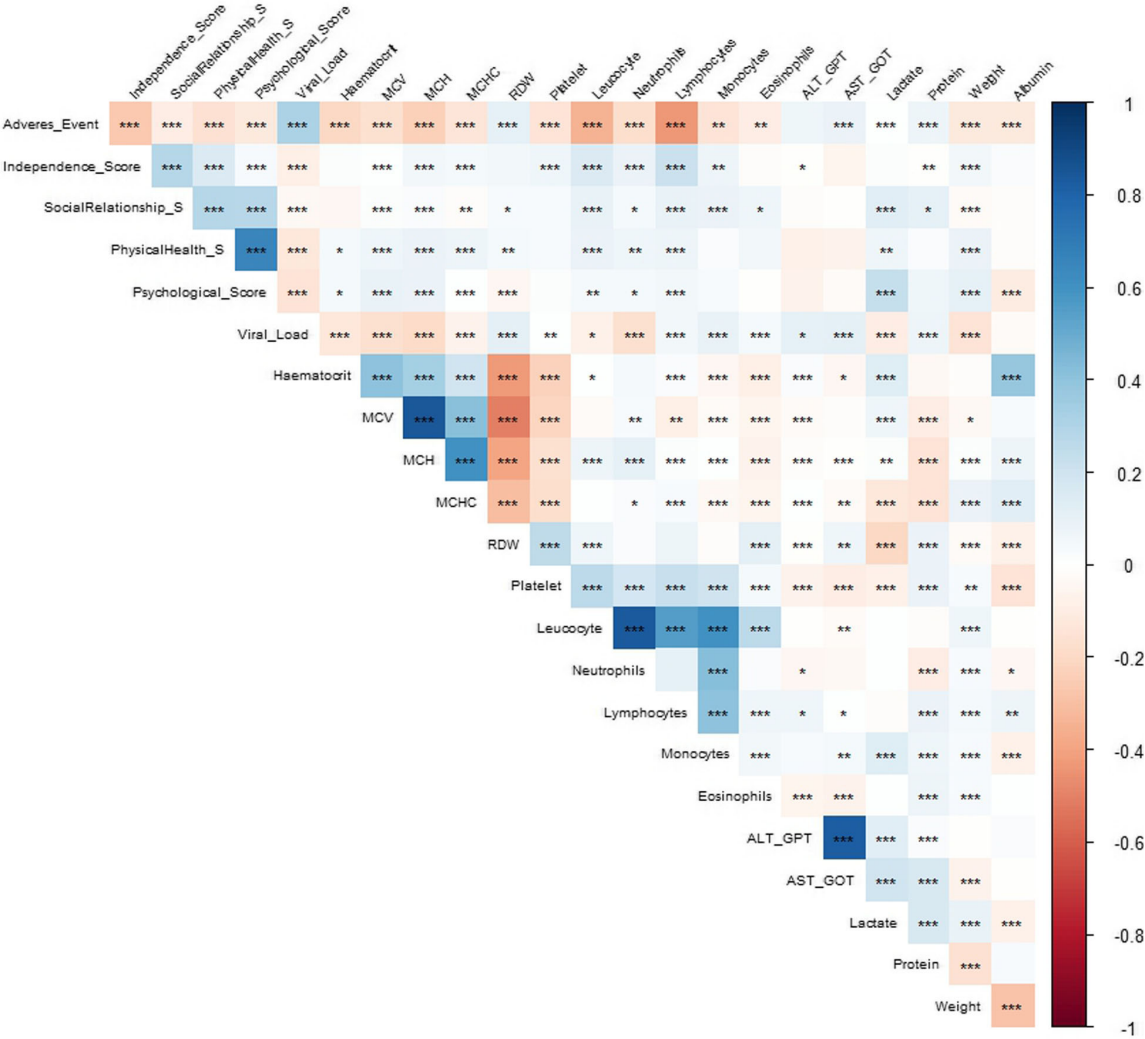
Univariable analysis: Correlogram of CD4 cell count, baseline QOL scores and hematological parameters. Blue indicates a positive correlation whereas red indicates a negative correlation. The symbols inside each square of the grid is statistical significance: (*) *P* < 0.05; (**) *P* < 0.01; (***) *P* < 0.00

The degree of severity had a negative correlation with lymphocyte count, MCHC, MCV, MCH, hematocrit levels, leukocyte counts, hemoglobin level (Hb), weight, serum albumin, and eosinophils. However, it had a positive correlation with viral load, total protein, LDH, ALT, AST and red cell distribution width (RDW) (see Fig. 1).

Demographic and clinical parameters were compared in the four grouping categories of CD4 counts (see supplementary Figure S1). Lower lymphocyte count, monocytes count, leukocyte count MCH, MCV, platelet count, neutrophils and hematocrit levels were associated with lower CD4 cells count (Figure S1(A-G, O)). Moreover, patients who were at a severe disease stage, further had a higher viral load, ALT and AST.

### Longitudinal change of adverse events

**Fig. 2:**
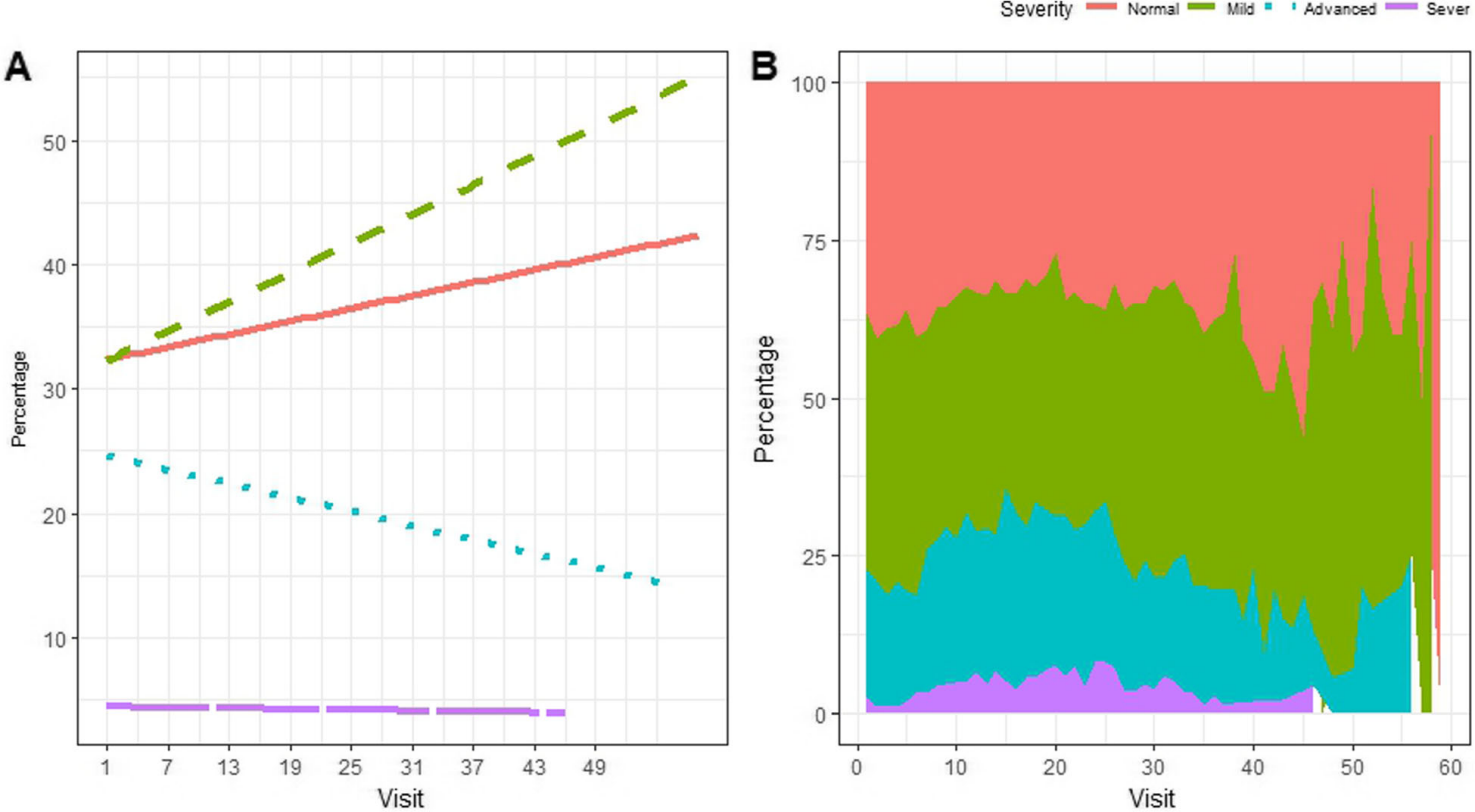
Individual profile plot using (**a**) Smoothed line plot, (**b**) Stacked plot

The profile plot presented in Fig. 2 displays the longitudinal change of CD4 count categories for HIV infected patients. From Fig. 2a, we note that the overall CD4 cell count had improved among HIV-infected patients in the long run. Patients with advanced, mild and normal stages of the disease, are indeed the most frequent categories at the beginning of the period. Then, some of the patients with advanced and severe stages of the disease had immunologically recovered after the treatment. Moreover, for further assessment of the longitudinal change of CD4 cell count, we plotted a stacked plot (Fig. 2b). Fig. 2b compliments our reflections from Fig. 2a.

### Factor analyses of clinical parameters

Considering white blood cell parameters, the first three components (factors) accounted for 77% of the observed variations. We assigned the names of these factors, based on the loadings that contributed most highly to each of the items, so that factor 1 is granulocytes components (which has high loading for leucocytes (0.925), neutrophils (0.936) and monocytes (0.635)), factor 2 is mononuclear components (which has high loading for the variables lymphocytes (0.838) and basophils (0.616)) and factor 3 is related to eosinophils. Similarly, considering red blood cell parameters, the first two components (factors) accounted for 81% of the observed variations and factor 1 related to hemoglobin and hematocrit, while factor 2 related to red blood cell indices. Considering blood chemistry parameters, the first two components (factors) accounted for 72% of the observed variations and factor 1 related to liver abnormality, while factor 2 related to electrolyte comp. Moreover, considering protein and lipids parameters, the first two components (factors) accounted for 65% of the observed variations and factor 1 related to lipids component, while factor 2 related to protein component (see Table 2).

**Table 2:** Clinical parameters and corresponding factor loadings from the rotated factors

Clinical parameters	Principal Components	Variables	Rotated factor loadings	Commutative variations
White blood cell parameters	1. Granulocytes component	Leucocyte	**0.925**	77%
		Neutrophils	**0.936**	
		Monocytes	**0.635**	
	2. Mononuclear component	Lymphocytes	**0.838**	
		Basophils	**0.616**	
	3. Eosinophils component	Eosinophils	**0.947**	
Red blood cell parameters	4. Hb and haematocrit component	RBC counts	**0.946**	81%
		Hb	**0.886**	
		Haematocrit	**0.919**	
	5. RBC indices component	MCV	**0.953**	
		MCH	**0.825**	
		MCHC	**0.521**	
		RDW	**−0.592**	
Blood chemistry	6. Liver enzyme abnormality component	ALT (GPT)	**0.829**	72%
		AST (GOT)	**0.967**	
	7. Electrolyte component	Chloride	**0.455**	
		Sodium	**0.994**	
		Calcium	**0.213**	
Protein and lipids	8. Lipid component	Cholesterol	**0.971**	65%
		LDL	**0.917**	
		Triglycerides	**0.360**	
	9. Protein Comp	LDH	**−0.769**	
		Total protein	**0.670**	

## Results of multilevel ordinal models

Table 3 presents the results of both multilevel ordinal proportional-odds and non-proportional-odds-model. Both models included a time effect (per month) and covariates effect. However, comparing log-likelihood statistic clearly rejects the proportional-odds-assumption (log-likelihood ratio χ^2^ = 159.51) indicating that the effects of the predictor covariates can’t be assumed identical across the three cumulative logits. So, we used a multilevel ordinal non-proportional odds model to examine the long-time trend of adverse events of HIV infected patients. Moreover, the deviance information criteria also showed multilevel growth ordinal non-proportional odds model fits better for our dataset used.

**Table 3:** Estimates and the 95% confidence intervals for parameters of multilevel ordinal non-proportional-odds model

Effect	Non-Proportional Odds		
	Normal vs Mild, Advance, Severe	Normal, Mild vs Advance, Severe	Normal, Mild, Advance vs Severe
	Exp (β) (95%CI)	Exp (β) (95%CI)	Exp (β) (95%CI)
**Thresholds**			
Normal	0.02 (0.01, 0.09)*		
Mild		19.87 (3.71, 50.4)*	
Advanced			24.51 (3.62, 81.35)*
Age: 21–39 years	**3.62 (1.03, 10.41**)**	3.36 (1.64, 36.99)*	4.15 (0.99, 50.32)
Age: 40–59 years	2.24 (0.75, 7.35)	1.58 (0.44, 4.81)	3.23 (0.82, 20.53)
Education: Grade 9–10	1.06 (0.17, 5.31)	0.72 (0.10, 3.77)	2.28 (0.17, 19.40)
Education: Grade ≥11	2.13 (1.05, 12.11)*	2.07 (0.54, 11.26)	3.61 (1.03, 35.21)*
Sex while Drunk: No	3.34 (1.22, 10.63)**	5.21 (1.71, 17.08)*	2.57 (0.57, 13.71)
Weight	1.01 (0.99, 1.03)	1.02 (1.01, 1.03)*	1.02 (0.98, 1.03)
Marital status: Single/no sexual partners	3.28 (0.71, 10.27)	3.45 (0.97, 10.48)	0.57 (0.07, 5.31)
Marital status: Many sexual partners	0.88 (0.14, 4.95)	0.19 (0.01, 2.20)	0.12 (0.01, 0.19)*
cART: After initiation	1.42 (0.60, 3.33)	1.27 (1.11, 1.45)**	1.28 (1.15, 1.43)**
Physical health score	1.10 (0.99, 1.17)	1.11 (1.07, 1.18)*	1.57 (1.41, 1.62)**
Level of independence scores	1.18 (1.12, 1.27)*	1.02 (0.96, 1.18)	0.83 (0.71, 1.01)
Social relationship scores	0.96 (0.90, 1.01)	0.93 (0.88, 0.98)	0.92 (0.78, 1.11)
Psychological well-being scores	0.94 (0.81, 1.11)	0.77 (0.71, 1.81)	0.51 (0.47, 1.68)
RBC indices component	1.06 (0.91, 1.23)	0.96 (0.82, 1.10)	0.85 (0.55, 1.30)
Electrolyte component	1.11 (1.04, 1.29)*	1.09 (1.01, 1.18)*	0.99 (0.77, 1.27)
Mononuclear component	1.73 (1.48, 2.05)*	1.79 (1.51, 2.12)*	2.14 (0.98, 3.49)
Viral Load	**0.52 (0.41, 0.66**)*	0.56 (0.46, 0.67)*	0.44 (0.32, 0.82)*
**Time Slope**			
Visit	0.55 (0.49, 1.06)	0.66 (0.61, 1.35)	1.76 (1.53, 1.94)*
cART: After initiation	1.62 (1.34, 1.91)***	1.67 (1.39, 2.01)**	2.10 (1.45, 3.04)**
Physical health score	1.02 (1.01, 1.03)**	1.02 (1.01, 1.03)**	1.03 (0.99, 1.05)
Psychological well-being scores	1.01 (0.99, 1.02)	1.02 (1.01, 1.03)**	1.02 (1.01, 1.03)**
Level of independence scores	1.02 (1.01, 1.04)*	1.02 (1.01, 1.04)*	1.02 (1.01, 1.04)*
Social relationship scores	1.01 (0,99, 1.01)	1.01 (1.003, 1.02)*	1.01 (0.99, 1.02)
Education: Grade 9–10	1.02 (0.97, 1.10)	1.04 (0.98, 1.12)	0.99 (0.91, 1.11)
Education: Grade ≥11	1.03 (1.01, 1.07)*	1.10 (1.05, 1.15)**	1.01 (0.96, 1.06)
Age: 21–39 years	1.15 (1.01, 1.29)*	1.04 (1.01, 1.07)*	1.02 (0.94, 1.06)
Age: 40–59 years	1.04 (0.97, 1.09)	1.02 (0.96, 1.09)	1.11 (0.92, 1.42)
Marital status: Single/no sexual partners	0. 94 (0.89, 0.95)*	0.95 (0.88, 1.07)	1.02 (0.92, 1.09)
Marital status: Many sexual partners	0.91 (0.84, 0.99)*	0.96 (0.94, 0.99)*	1.07 (0.93, 1.27)
Sex while drunk: No	0.94 (0.92, 1.04)	1.04 (0.99, 1.08)	1.03 (0.97, 1.06)
Viral load	1.01 (0.99, 1.02)	1.01 (0.99, 1.03)	0.96 (0.93, 0.99)*
Weight	1.00 (0.99, 1.01)	1.00 (0.99, 1.01)	1.01 (0.99, 1.02)
Electrolyte component	1.02 (1.003, 1.07)*	1.01 (1.001, 1.04)*	1.00 (0.99, 1.02)
RBC indices component	1.01 (0.99, 1.02)	1.02 (1.01, 1.03)*	1.02 (0.99, 1.04)
Mononuclear component	1.01 (0.99, 1.03)	0.99 (89, 1.01)	0.99 (0.97, 1.03)
**Random components of non-proportional odds**			
Intercepts variance	12.21* **(9.96, 15.12)**		
interpt-slope covariance	−0.36* **(−0.49, − 0.22)**		
Variance (Time slope)	0.02* **(0.01, 0.02)**		
**Model Diagnosis**			
	**Proportional Odds**	**Non-Proportional Odds**	
−2 log L	8656.51	8426.78	
Bayesian DIC	9732.43	9301.43	
Deviance (dbar)	9367.12	9221.87	

Keys:- Statistical significance: (*) *P* < 0.05; (**) *P* < 0.01; (***) *P* < 0.001; Reference category: ART [Before initation]; Age [≤20 years]; Education [≤8 grade]; Marital status [Married]; Sex while drunk [Yes]

Since our outcome has four categories, the model was set up using 3 cumulative logit models. The first logit model was normal (no adverse) versus mild, advanced or severe immunological stages (category 1 versus 2, 3 and 4) that can be interpreted as the odds of a patient being in the “normal” immunological stage, given that the values of covariates. The second cumulative logit model was normal or mild versus advanced or severe immunological stage (category 1and 2 versus 3 and 4) that can be interpreted as the odds of a patient being in the “mild or normal” immunological stage at time *t*, given that the values of covariates. Moreover, the final logit model was normal, mild or advance versus severe immunological stage (category 1, 2 and 3 versus 4) given the values of the independent variables, the probability of patient *j* being in the “normal, mild or advance” immunological stage at a given time.

Results of the generalized multilevel models in Table 3 showed age has a significant effect on the odds of patients being in the “normal” immunological stage (aOR = 3.62; 95%CI: 1.03–10.41), indicating that middle-aged patients are significantly associated with increased chances of being in the “normal” immunological stage as compared to that of younger age. Similarly, middle-aged patients are significantly associated with increased chances of being in the “normal or mild” immunological stage (aOR = 3.36; 95%CI: 1.64–36.99). In addition, age has a differential effect across categories but the direction of the effects are the same for both “normal” and “normal or mild” immunological stages. Patients with higher education levels are significantly associated with the odds of being in the “normal” (aOR = 2.13; 95%CI: 1.05–12.11) and “normal, or mild” immunological stage (aOR = 3.61; 95%CI: 1.03–35.21). The effects of educational level are larger for a patient being in the “normal or mild” immunological stage than that of a patient being in the “normal” immunological stage. Patients who did not have sex under the influence of alcohol are significantly associated with being in the “normal” (aOR = 3.34; 95%CI: 1.22–10.63) and “normal or mild” immunological stage (aOR = 5.21; 95%CI: 1.71–17.08). Moreover, increases in weight increase the likelihood of a patient being in the “normal or mild” immunological stage. Patients who reported many sex partners are significantly associated with lower chances of being in the “normal, mild and advance” immunological stage (aOR = 0.12; 95%CI: 0.01–0.19), compared to those who reported having stable sex partners.

Among the control variables, cART initiation has a significant effect on a patient being in the “normal or mild” (aOR = 1.27; 95%CI: 1.11–1.45) and “normal, mild and advance” immunological stage (aOR = 1.28; 95%CI: 1.15–1.43). Considering quality of life variables, patients having a high physical health scores were associated with increased chances of being in the “normal or mild” (aOR = 1.11; 95%CI: 1.07–1.18) and “normal, mild or advance” immunological stage (aOR = 1.57; 95%CI: 1.41–1.62). The effects of physical health scores are larger for the patient being in the “normal, mild or advance” than that of a patient being in the “normal or mild” immunological stage. Similarly, with regard to hematological parameters, higher scores of electrolyte latent and higher mononuclear scores had a significant effect on better immunological stages (particularly “normal” and “normal or mild”). Moreover, increases in viral load decrease the likelihood of a patient being in better CD4 count categories.

Considering the time effects of the covariates, there is a statistically significant linear trend for visits (comparing normal, mild or advance combined versus severe immunological stage) (aOR = 1.76; 95%CI: 1.53–1.94), indicating that the log odds of being in “normal, mild or advance” immunological stage is increasing with the follow-up time. Compared to before cART initiation, the likelihood of a patient being in the “normal” (aOR = 1.62; 95%CI: 1.34–1.91), “normal or mild” (aOR = 1.67; 95%CI: 1.39–2.01) and “normal, mild or advance” immunological stages (aOR = 2.10; 95%CI: 1.45–3.04) after cART initiation is increasing with time. In addition, cART initiation has a differential effect across the CD4 immunological categories.

Considering quality of life domain scores, patients with higher physical health scores are associated with increased chances of being in “normal” (aOR = 1.02; 95%CI: 1.01–1.07) and “normal or mild” immunological stage (aOR = 1.02; 95%CI: 1.01–1.03) with time. Furthermore, an increase in psychological well-being scores increases the likelihood of patients being in the “normal or mild” (aOR = 1.02; 95%CI: 1.01–1.03) and “normal, mild or mild” immunological stage (aOR = 1.02; 95%CI: 1.01–1.03)) with time. Similarly, an increase in the level of independence scores increases the likelihood of a patient being in better immunological stages (particularly “normal or mild” and “normal, mild or mild”). In addition, there was no differential effects of QoL domain scores across immunological categories.

Patients with higher educational levels were significantly associated with increased chances of being in the “normal” (aOR = 1.03; 95%CI: 1.01–1.07) and “normal or mild” immunological stage (aOR = 1.10; 95%CI: 1.05–1.15) through the follow-up time. The effects of educational level are larger for a patient being in the “normal or mild” immunological stage than that of a patient being in the “normal” immunological stage. As the follow-up time progresses, patients in middle-aged group (21-39 years) were more likely to have a better immunological stage (particularly “normal” or “normal and mild”) compared to those younger patients. Patients who reported many sex partners are significantly associated with decreased chances of being in the “normal” (aOR = 0.91; 95%CI: 0.84–0.99) and “normal or mild” immunological stage (aOR = 0.96; 95%CI: 0.94–0.99) with time, as compared to patients with stable partners. Moreover, through the follow-up time, as RBC indices and electrolyte scores increase the likelihood of a patient being in better immunological stages increases (particularly “normal or mild”).

Finally, considering the variance components for the model, it may be seen that considerable variation remains in the intercepts (${\sigma}_{v_0}^2$ =12.81, *p* < .05), as well as in the slopes (${\sigma}_{v_1}^2$ =0.02, *p* < .05). Moreover, the intra-class correlation at a person-level equals 0.71 and 0.76 for multilevel growth ordinal proportional-odds and non-proportional-odds models respectively. This is a large ICC, so we can justify that multilevel ordinal analysis is appropriate for this HIV/AIDS cohort data. (See Table 3).

## Discussion and conclusion

The multilevel ordinal proportional-odds model offers a particularly attractive approach to the analysis of longitudinal ordinal responses [34–36]. When the proportionality assumption is not satisfied, the non-proportional-odds model will often be a desirable alternative. The benefit of multilevel ordinal model is that they account for floor and ceiling effects of the outcome variable as compared to linear mixed effect models [4]. In particular, when an outcome variable is highly skewed, which is usually the case in clinical and epidemiological studies, setting the response into the ordinal scale is advantageous. Furthermore, as noted [37], the multilevel non-proportional-odds model is a parsimonious approach by permitting the covariates that meet proportional odds assumption to take the same coefficient for all cumulative logit models, and other covariates to vary between cumulative logit models, thus ensuring no potential loss in accuracy of prediction. Also, using a multilevel non-proportional-odds model for treatment provided a more informative analysis than using a proportional odds model [38]. A further advantage of the multilevel non-proportional-odds model is that it provides more specific information about the differential effect of covariates on the outcome. However, the interpretation and justification for the non-proportional odds are less straight forward than it is for the multilevel proportional odds model.

Patients with higher educational levels tend to have better immunological stages (particularly “normal” or “normal and mild”) through the follow-up time. We further observed that educational level has a differential effect across immunological stages but the direction of the educational level’s effect is the same for both “normal” and “normal and mild” immunological stages. Our finding is concurrent with those from prior reports [15, 39], which noted that patients having higher educational level significantly associated with a better rate of change of immunological recovery. This might be due to literate patients having financial resources, work capacity and access to quality health and social care. Patients who reported many sex partners tend to have a worsen immunological stage (particularly “normal”) with time, compared to patients with stable partners. As has been previously shown [40], patients with higher incidences of sexual risk-taking behavior (such as many sex partners) were significantly associated with low QoL and chronic depression of HIV patients. Chronic depression and low QoL scores are significantly linked to lower CD4 cell count [41, 42], showing that the effect of many sex partners on incomplete immune recovery, is mediated through depression and QoL.

Patients in the middle-aged group (21–39 years) were significantly associated with increased chances of being in better immunological stages (particularly “normal” or “normal and mild”) through the follow-up time. In particular, the effects of age are larger for a patient being in the “normal and mild” immunological stage than that of a patient being in the “normal” immunological stage. Previous studies [43, 44] also reported that middle-aged adults experienced higher rates of CD4 recovery. Contrary to our findings, an inverse relationship between the age of the patient and CD4 count recovery has been reported in previous studies [45–47]. That is, younger age was associated with a better rate of change of immunological recovery. Furthermore, the likelihood of a patient being in better immunological stages after cART initiation is increasing with time, which is in agreement with findings reported in [48, 49]. We further observed that cART initiation has a differential effect across immunological stages.

We have also found that patients with high *scores* in *quality* of *life* were more likely to have better immunological stages (particularly “normal and mild”) through the follow-up time. This was supported by studies in South Africa, Venter et al. [50] and Ingumbor et al. [51]. These two studies have found a significant positive association between trends of CD4 count and health-related QoL scores. In contrast, a cross-sectional study in Uganda showed a weak positive correlation between QoL domain scores and CD4 count [52]. Possible explanations for this controversial report might be that data for our study was conducted in a cohort of acutely infected patients and followed up repeatedly over an extended ART-free period.

Among the different clinical attributes of patients, patients having high scores of latent variable related to calcium, chloride, and sodium in the blood were more likely to have a better immunological stage (particularly “normal” or “normal and mild”) through the follow-up time, which is in agreement with findings reported in [53–55]. We further noted that patients having high scores of RBC indices significantly improves the rate of change of immunological recovery of HIV/AIDS patients with time. Our findings are concurrent with those from prior studies [54–56], which observed that a positive correlation exists between RBC indices (such as MCH and MCV) and CD4 cell count. Thus, hematological parameters such as electrolyte components (i.e. chloride, sodium, and calcium) and RBC indices (i.e. MCH and MCV) could thus help health workers identify individuals with poor immunological and clinical responses in the absence of CD4 cell count.

There are some limitations regarding the multilevel non-proportional-odds model, including the choice of a specific link function. Although we focused on the cumulative logit link function in the current study, this is only one of several possible link functions one may use within the context of the non-proportionality constraints. We did not evaluate model performance with alternative link functions, such as cauchit, probit, and complementary log-log. However, model diagnostics have been performed and the residual and influence diagnostics affirmed no violation of implicit and explicit assumptions in our model.

Finally, from a methodological perspective, it can be concluded that multilevel non-proportional-odds approach provides a flexible modeling alternative when the proportional-odds assumption of equal effects of the predictor variables at every stage of the response variable is violated. Moreover, it provided more specific information about the effect of covariates on the outcome. From clinical results, patients having higher educational levels, higher QoL scores, higher RBC indicies and stable sex partner were found to be the predicting factors for trends of CD4 count immunological recovery.

## Supplementary information

**Additional file 1: Figure S1.** Box plots of hematological parameters (A) Neutrophil, (B) MCV, (C) Haematocrit level, (D) Platelet Count, (I) Leucocyte count, (F) Lymphocyte count, (G) Monocyte, (H) Eosinophils, (I) protein, (J) Viral load, (K) ALT, (L) AST, (M) Weight, (N) RDW and (O) MCH according to immunological stage of HIV/AIDS.

## Data Availability

The dataset used and analyzed during the current study is available from the corresponding author on reasonable request.

## Abbreviations

*AIDS*: Acquired Immune Deficiency Syndrome
ALT: Alanine aminotransferase
ART: Antiretroviral therapy
*ARV*: Antiretroviral drug
AST: Aspartate aminotransferase
BP: Blood pressure
CAPRISA: Center of the AIDS program of research in South Africa
HB: Hemoglobin
HIV: Human Immunodeficiency Virus
LDH: Lactate dehydrogenase
MCH: Mean corpuscular hemoglobin
MCHC: Mean corpuscular hemoglobin concentration
MCV: Mean corpuscular volume
OI: *Opportunistic infections*
PLHIV: people living with HIV
PR: Pulse rate
QoL: Quality of life
RBC: Red blood cells
RDW: Red cell distribution width
V B12: Vitamin B12

## References

[1] Hoffman J, Van Griensven J, Colebunders R, McKellar M (2010). Role of the CD4 count in HIV management. HIV Ther.

[2] Langford SE, Ananworanich J, Cooper DA (2007). Predictors of disease progression in HIV infection: a review. AIDS Res Ther.

[3] Hedeker D (2015). Methods for multilevel ordinal data in prevention research. Prev Sci.

[4] Winship C, Mare RD (1984). Regression models with ordinal variables. Am Sociol Rev.

[5] McKelvey RD, Zavoina W (1975). A statistical model for the analysis of ordinal level dependent variables. J Math Sociol.

[6] Braunstein SL, Robertson MM, Myers J, Abraham B, Nash D (2016). Increase in CD4+ T-cell count at the time of HIV diagnosis and antiretroviral treatment initiation among persons with HIV in new York City. J Infect Dis.

[7] Kulkarni H, Okulicz JF, Grandits G, Crum-Cianflone NF, Landrum ML, Hale B (2011). Early Postseroconversion CD4 cell counts independently predict CD4 cell count recovery in HIV-1–Postive subjects receiving antiretroviral therapy. J Acquir Immune Defic Syndr (1999).

[8] Mu Y, Wei Y (2009). A dynamic quantile regression transformation model for longitudinal data. Stat Sin.

[9] Zhang H, Huang Y, Wang W, Chen H, Langland-Orban B (2017). Bayesian quantile regression-based partially linear mixed-effects joint models for longitudinal data with multiple features. Stat Methods Med Res.

[10] Adams M, Luguterah A (2013). Longitudinal analysis of change in CD4+ cell counts of HIV-1 patients on antiretroviral therapy (ART) in the Builsa district hospital. Eur Sci J.

[11] Chakraborty H, Iyer M, Duffus WA, Samantapudi AV, Albrecht H, Weissman S (2015). Disparities in viral load and CD4 count trends among HIV-infected adults in South Carolina. AIDS Patient Care STDs.

[12] De La Mata NL, Ly PS, Ng OT, Nguyen KV, Merati TP, Pham TT (2017). Trends in CD4 cell count response to first-line antiretroviral treatment in HIV-positive patients from Asia, 2003–2013: TREAT Asia HIV observational database low intensity transfer. Int J STD AIDS.

[13] Gezie LD (2016). Predictors of CD4 count over time among HIV patients initiated ART in Felege Hiwot referral hospital, Northwest Ethiopia: multilevel analysis. BMC Res Notes.

[14] Montarroyos UR, Miranda-Filho DB, César CC, Souza WV, Lacerda HR (2014). Albuquerque MdFPM, Aguiar MF, de Alencar Ximenes RA. Factors related to changes in CD4+ T-cell counts over time in patients living with HIV/AIDS: a multilevel analysis. PLoS One.

[15] Seyoum A, Temesgen Z (2017). Joint longitudinal data analysis in detecting determinants of CD4 cell count change and adherence to highly active antiretroviral therapy at Felege Hiwot teaching and specialized hospital, North-West Ethiopia (Amhara region). AIDS Res Ther.

[16] Trotta MP, Cozzi-Lepri A, Ammassari A, Vecchiet J, Cassola G, Caramello P (2010). Rate of CD4+ cell count increase over periods of viral load suppression: relationship with the number of previous virological failures. Clin Infect Dis.

[17] Goldstein H. Multilevel Statistical Models. 4th ed. New York: Wiley; 2010.

[18] van Loggerenberg F, Mlisana K, Williamson C, Auld SC, Morris L, Gray CM (2008). Establishing a cohort at high risk of HIV infection in South Africa: challenges and experiences of the CAPRISA 002 acute infection study. PLoS One.

[19] Garrett N, Norman E, Leask K, Naicker N, Asari V, Majola N (2018). Acceptability of early antiretroviral therapy among south African women. AIDS Behav.

[20] Karim QA, Karim SSA, Frohlich JA, Grobler AC, Baxter C, Mansoor LE (2010). Effectiveness and safety of tenofovir gel, an antiretroviral microbicide, for the prevention of HIV infection in women. Science.

[21] Dessie ZG, Zewotir T, Mwambi H, North D (2020). Modelling of viral load dynamics and CD4 cell count progression in an antiretroviral naive cohort: using a joint linear mixed and multistate Markov model. BMC Infect Dis.

[22] Dessie ZG, Zewotir T, Mwambi H, North D (2020). Modeling viral suppression, viral rebound and state-specific duration of HIV patients with CD4 count adjustment: parametric multistate frailty model approach. Infect Dis Ther.

[23] Organization WH (2007). WHO case definitions of HIV for surveillance and revised clinical staging and immunological classification of HIV-related disease in adults and children.

[24] Dessie ZG, Zewotir T, Mwambi H, North D (2020). Modelling immune deterioration, immune recovery and state-specific duration of HIV-infected women with viral load adjustment: using parametric multistate model. BMC Public Health.

[25] Byrne BM (2005). Factor analytic models: viewing the structure of an assessment instrument from three perspectives. J Pers Assess.

[26] Kaiser HF (1958). The varimax criterion for analytic rotation in factor analysis. Psychometrika..

[27] Agresti A (1996). An introduction to categorical data analysis.

[28] O’Connell AA (2006). Logistic regression models for ordinal response variables: sage.

[29] Bender R, Benner A (2000). Calculating ordinal regression models in SAS and S-plus. Biom J.

[30] Fielding A, Yang M, Goldstein H (2003). Multilevel ordinal models for examination grades. Stat Model.

[31] O’Connell AA, Doucette HL (2007). Modeling longitudinal ordinal response variables for educational data. J Mod Appl Stat Methods.

[32] Omar RZ, Thompson SG (2000). Analysis of a cluster randomized trial with binary outcome data using a multi-level model. Stat Med.

[33] Hedeker D, Mermelstein RJ (1998). A multilevel thresholds of change model for analysis of stages of change data. Multivar Behav Res.

[34] Agresti A. Categorical data analysis. 2nd ed. New York: Wiley; 2003.

[35] Long SJ, Long JS, Freese J (2006). Regression models for categorical dependent variables using Stata: Stata press.

[36] McCullagh P (1980). Regression models for ordinal data. J Royal Stat Soc Series B (Methodological).

[37] Sasidharan L, Menendez M (2014). Partial proportional odds model—an alternate choice for analyzing pedestrian crash injury severities. Accid Anal Prev.

[38] Liu LC, Hedeker D (2006). A mixed-effects regression model for longitudinal multivariate ordinal data. Biometrics..

[39] Jiang H, Xie N, Cao B, Tan L, Fan Y, Zhang F (2013). Determinants of progression to AIDS and death following HIV diagnosis: a retrospective cohort study in Wuhan, China. PLoS One.

[40] Vu T, Boggiano V, Tran B, Nguyen L, Tran T, Latkin C (2018). Sexual risk behaviors of patients with HIV/AIDS over the course of antiretroviral treatment in northern Vietnam. Int J Environ Res Public Health.

[41] Ickovics JR, Hamburger ME, Vlahov D, Schoenbaum EE, Schuman P, Boland RJ (2001). Mortality, CD4 cell count decline, and depressive symptoms among HIV-seropositive women: longitudinal analysis from the HIV epidemiology research study. Jama..

[42] Rivera-Rivera Y, Vázquez-Santiago FJ, Albino E, MdC S, Rivera-Amill V (2016). Impact of depression and inflammation on the progression of HIV disease. J Clin Cell Immunol.

[43] Saracino A, Zaccarelli M, Lorenzini P, Bandera A, Marchetti G, Castelli F (2018). Impact of social determinants on antiretroviral therapy access and outcomes entering the era of universal treatment for people living with HIV in Italy. BMC Public Health.

[44] Wong NS, Chan KCW, Cheung EKH, Wong KH, Lee SS (2017). Immune recovery of middle-aged HIV patients following antiretroviral therapy: an observational cohort study. Medicine.

[45] Zhou J, Sirisanthana T, Kiertiburanakul S, Chen Y-MA, Han N, Lim PL (2010). Trends in CD4 counts in HIV-infected patients with HIV viral load monitoring while on combination antiretroviral treatment: results from the TREAT Asia HIV observational database. BMC Infect Dis.

[46] Ledergerber B, Collaboration P (2004). Predictors of trend in CD4-positive T-cell count and mortality among HIV-1-infected individuals with virological failure to all three antiretroviral-drug classes. Lancet.

[47] Egger S, Petoumenos K, Kamarulzaman A, Hoy J, Sungkanuparph S, Chuah J (2009). Long-term patterns in CD4 response is determined by an interaction between baseline CD4 cell count, viral load and time: the Asia Pacific HIV observational database (APHOD). J Acquir Immune Defic Syndr (1999).

[48] Kiertiburanakul S, Boettiger D, Lee MP, Omar SF, Tanuma J, Ng OT (2014). Trends of CD4 cell count levels at the initiation of antiretroviral therapy over time and factors associated with late initiation of antiretroviral therapy among Asian HIV-positive patients. J Int AIDS Soc.

[49] Lok JJ, Bosch RJ, Benson CA, Collier AC, Robbins GK, Shafer RW (2010). Long-term increase in CD4+ T-cell counts during combination antiretroviral therapy for HIV-1 infection. AIDS (London, England).

[50] Venter E, Gericke GJ, Bekker P (2009). Nutritional status, quality of life and CD4 cell count of adults living with HIV/AIDS in the Ga-Rankuwa area (South Africa). South Afr J Clin Nutr.

[51] Ingumbor J, Steward A, Holzemer W (2013). Comparison of the health related quality of life, CD4 count and viral load of AIDS patients with HIV who have been on treatment for 12 months in rural South Africa.

[52] Mwesigire DM, Martin F, Seeley J, Katamba A (2015). Relationship between CD4 count and quality of life over time among HIV patients in Uganda: a cohort study. Health Qual Life Outcomes.

[53] Janbakhsh A, Mansouri F, Vaziri S, Sayad B, Afsharian M, Miladi R (2018). Serum levels of vitamin D, magnesium, calcium, iron, and TIBC in HIV-infected patients. J Kerman Univ Med Sci.

[54] Tinarwo P, Zewotir T, North D (2019). Covariate random effects on the CD4 count variation during HIV disease progression in women. HIV/AIDS (Auckland, NZ).

[55] Tinarwo P, Zewotir T, Yende-Zuma N, Garrett NJ, North D (2019). An evaluation to determine the strongest CD4 count covariates during HIV disease progression in women in South Africa. Infect Dis Ther.

[56] Lumbanraja SN, Siregar D. Association between red blood cell indices and CD4 count in HIV-positive reproductive women. IOP Conference Series: Earth and Environmental Science. 2018;125(1):012027.

