# Supplementary figures and images for: Multilevel ordinal model for CD4 count trends in seroconversion among South Africa women
Source: BMC Infect Dis. 2020 Jun 23;20:447. doi: 10.1186/s12879-020-05159-4 (PMC7310392; doi:10.1186/s12879-020-05159-4)

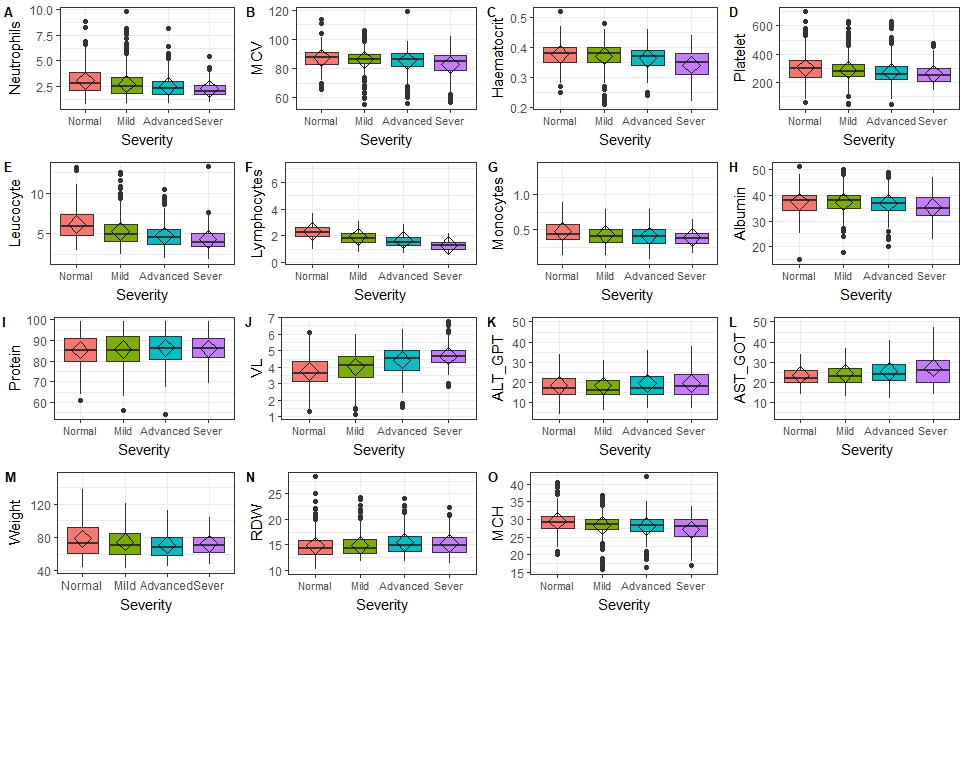

Supplement: Supplementary file 1 — Additional file 1: Figure S1. Box plots of hematological parameters (A) Neutrophil, (B) MCV, (C) Haematocrit level, (D) Platelet Count, (I) Leucocyte count, (F) Lymphocyte count, (G) Monocyte, (H) Eosinophils, (I) protein, (J) Viral load, (K) ALT, (L) AST, (M) Weight, (N) RDW and (O) MCH according to immunological stage of HIV/AIDS. [file 12879_2020_5159_MOESM1_ESM.jpeg]
